# Inter- and intra-rater reliability of new application software for computerised paediatric version of Wisconsin Gait Scale

**DOI:** 10.1038/s41598-023-31436-8

**Published:** 2023-03-23

**Authors:** Agnieszka Guzik, Andżelina Wolan-Nieroda, Mariusz Drużbicki

**Affiliations:** Department of Physiotherapy, Institute of Health Sciences, College of Medical Sciences, University of Rzeszów, Rzeszów, Poland

**Keywords:** Movement disorders, Paediatric neurological disorders

## Abstract

The paediatric version of Wisconsin Gait Scale (WGS) is a reliable tool for gait assessment in children with spastic hemiplegic cerebral palsy (CP). We decided to develop a solution which will make it possible to objectify the descriptive paediatric version of the WGS, and which, consequently, will allow researchers/clinicians to more easily perform accurate assessment of gait patterns in patients. The aim of the study was to assess inter- and intra-rater reliability of new application software for computerised paediatric version of the WGS in children with hemiplegic CP. The study involved 31 children with hemiplegic CP. The app was designed using a model based on thematic categories of the paediatric WGS, and utilising auxiliary lines between specific points on the patient’s body, and taking into account angular values, duration and length of the specific gait phases, in order to enable acquisition of quantitative data corresponding to the components of the WGS. The gait of the study participants was recorded, in series of videos. These provided material for three independent raters who reviewed the recordings twice and assessed the participants’ gait using the app. After the evaluation was completed, the data were retrieved from the software. The new application software for the computerised paediatric WGS presents very good inter- and intra-rater reliability. Intra-class correlation coefficient (ICC) was very high in measurement 1 (ICC > 0.9) and 2 (ICC > 0.8), which reflects a very high degree of agreement between the three examiners; there was also high agreement for the specific examiners, between the two measurements (ICC > 0.9). The observational gait scale, objectified through the new software, and enabling computer-aided use of the paediatric WGS, presents practical advantages for examiners since it facilitates decisions taken in the process of WGS-based assessment in children with spastic hemiplegic CP.

## Introduction

Gait analysis is a standard component of comprehensive assessment of children with cerebral palsy (CP), since walking skills are essential for daily life and for social participation and, consequently, they are commonly seen as one of the most important abilities related to various aspects of daily living^1,2^. For this reason, gait analysis in children with CP is in fact more effective than any type of static physical examination, since it makes it possible to identify functional deviations. What is more, it provides information necessary in formulating physiotherapeutic diagnosis, in evaluating effects of specific therapeutic interventions, and finally, in measuring rehabilitation outcomes^2–5^.

Instrumented gait analysis (IGA), providing objective, accurate and highly reliable information in the three planes of movement, has generally been approved as the gold standard for evaluation of walking skills^5–7^. However, IGA is based on novel technologies and instruments, and as a result it presents certain disadvantages from the viewpoint of daily clinical practice. In addition to the high economical cost and the resulting insufficient availability of the related tools for medical professionals, IGA is a complex time-consuming process requiring high level of expertise^4–6,8–10^. Due to the above, observational gait assessment is far more viable and more commonly used in many clinical settings than IGA, because of the low cost, speed and simple procedures involved^5,7,8,11–13^.

Usefulness of the observational Wisconsin Gait Scale (WGS) has been investigated by many researchers who have reported very good psychometric properties, including reliability, validity, and responsiveness of the tool when it was used as intended, to assess adult stroke patients for both gait pattern deviations and effectiveness of interventions^14–20^. Since gait patterns commonly observed in children with hemiplegic CP are very similar to those presented by adults with hemiplegia after stroke, we thought it justifiable to adapt the WGS for use in assessment of children with CP^21^. Indeed, in the related study, we showed that the WGS effectively enables evaluation of walking skills in children with spastic hemiplegic cerebral palsy (SHCP)^21^. As for the development procedure used in that case, at the first stage, the applicability of WGS items was assessed by reference to video recordings of gait in children with hemiplegic CP, and discussed in detail by a team of experts; as a result two items were modified to enable more accurate assessment of gait in this population. At the second stage, the same video recordings were rescored by the same raters, in line with the modified version. The new paediatric WGS ultimately differs from the original version in the scoring of the items related to weight shift to affected side, and knee flexion from toe off to mid swing. The tool was found with very good inter- and intra-rater reliability. Hence, this modified, simple, easy-to-use ordinal WGS appears to be effective as an additional tool to facilitate qualitative observational gait assessment in children with SHCP. The added value of the paediatric version of WGS, compared to other scales designed for observational gait assessment in children with CP, lies in the fact that it enables gait assessment in all the planes, and in terms of spatiotemporal and kinematic parameters^21^.

At present, there are no computerised tools for objectivised interpretation of observational information acquired, for the purpose of gait assessment, with the use of the paediatric version of WGS or with other scales enabling gait assessment in children with CP. The only research report available at present is related to the Edinburgh Visual Gait Score (EVGS), which enables complex evaluation of gait in children with CP^22^. The tool has been shown to present good psychometric properties, yet it poses certain disadvantages, since it requires a number of devices and is based on a complicated recording and measuring procedure. Due to the above, a smartphone video protocol was developed to facilitate the daily use of the EVGS in clinical settings^22^. Notably, unlike the paediatric version of WGS, the EVGS focuses exclusively on assessing kinematic gait parameters^23^ whereas the former tool enables multifactorial gait assessment, including all the planes as well as spatiotemporal and kinematic parameters specifically in children with SHCP^21^.

The above considerations provided motivation for our team to continue the related research and to develop a computerised tool which would objectify the descriptive paediatric version of the WGS, in order to aid the decision-making process and, consequently, improve usefulness of this specific scale in the clinical practice^21^. The solution discussed here was intended to enable structured and uniform assessment of specific gait patterns, based on a standardised template. The application software, developed by our team, incorporates in its design the components of the paediatric version of the WGS, and additionally utilises auxiliary lines between specific anthropometric points on the patient’s body, as well as measurements of angular values, and duration and length of the specific gait phases. This way the app enables acquisition of quantitative data corresponding to items of the paediatric WGS. As intended, this computer-aided observational assessment tool may provide support for clinicians in projections of clinical outcomes, and in the process of monitoring the effects of treatments administered to children with SHCP.

The aim of the study was to assess inter- and intra-rater reliability of the new application software for computerised paediatric version of the WGS in children with hemiplegic CP.

## Methods

### Participants

The study was conducted in a group of thirty-one children with SHCP (18 boys, 13 girls; aged 7–18 years). The Gross Motor Function Classification Scale (GMFCS) levels of the participants were as follows: 2 children were classified at level I, 28 children at level II, and 1 child at level III. The characteristics of the study participants are shown in Table 1. In addition to meeting the above basic inclusion criteria, the children enrolled for the study were able to walk a distance of 10 m without another person’s assistance (if necessary, they could use walking aids or Ankle–Foot Orthosis (AFO). Children who could not follow instructions, those with gait impairment linked to other neurologic or orthopaedic conditions and those subjected to any surgical interventions affecting lower limbs less than 6 months prior, were excluded from the study. The experimental protocol was approved by the local Bioethics Commission at the University of Rzeszow (Identifier: 30/01/2020). The study design complied with the Declaration of Helsinki. Parents or legal guardians of all the children, as well as the participants aged 16 or more, were informed about the purpose of the study and provided written consent for their inclusion in the study.

**Table 1:** Characteristics of the study participants.

Participant (N = 31)	
Sex (girls/boys), N	13/18
Hemiparesis (left/right), N	7/24
GMFCS levels (I/II/III/IV/V), N	(2/28/1/0/0)
Age (years), Mean (SD)	11.6 (2.1)
Weight (kg), Mean (SD)	139.7 (10.12)
Height (m), Mean (SD)	37.9 (7.89)
Gait speed (m/s), Mean (SD)	0.79 (0.32)

N—Number of participants, SD—standard deviation, GMFCS—Gross Motor Function Classification Scale.

### Measures

The new app dedicated to the modified paediatric version of the WGS enables assessment of 14 factors related to specific gait phases, i.e., stance, toe-off, swing, and heel strike (Table 2). Most of the 14 items are rated on a three-point scale, except for Item 1 and Item 11 which are rated on five-point and four-point scales, respectively. The potential overall scores are in the range from 13.35 to 42 points, a higher score reflecting poorer quality of the gait pattern. A total score is calculated from all the items. All the points acquired in items 2–10 and 12–14 are added, whereas the scores in Items 1 and 11 are weighted by 3/5 and 3/4, respectively, before they are added to the total score. The modified paediatric WGS differs from the original version, due to which options *a)* and *b)* are specified for the scores of 2 and 3 in item 4 (weight shift to affected side), and in item 11 (knee flexion from toe off to mid swing)^21^.

**Table 2:** Items of computerised paediatric WGS.

Stance phase affected leg 1. Use of hand-held gait aid	1 = No gait aid
	2 = Minimal gait aid use
	3 = Minimal gait aid, wide base
	4 = Marked use
	5 = Marked use, wide base
2. Stance time on affected side	1 = Equal
	2 = Unequal: compared to unaffected limb remains on the affected leg for a shorter period of time
	3 = Very Brief: least amount of time
3. Step length on unaffected side	1 = Step through
	2 = Foot does not advance beyond the toe of the affected foot
	3 = Step to behind or up to, but not beyond the affected foot
4. Weight shift to affected side	1 = Full shift
	2a = Decreased shift: head and trunk crosses midline, but not over the affected foot
	2b = Decreased shift: head and trunk crosses midline, but not over the unaffected foot, head and trunk for part of stance phase leaning towards the affected side
	3a = Very limited shift: head and trunk does not cross midline, minimal weight shift in the direction of the affected side
	3b = Very limited shift: head and trunk does not cross midline, minimal weight shift in the direction of the unaffected side, head and trunk during entire stance phase leaning towards the affected side
5. Stance width	1 = Normal: Up to one shoe width between feet
	2 = Moderate: Up to two shoe widths between feet
	3 = Wide: Greater than two shoe widths between feet
Toe off affected leg 6. Guardedness (pause prior to advancing affected leg)	1 = None: Good forward momentum with no hesitancy noted
	2 = Slight: Slight pauses prior to toe off
	3 = Marked hesitation: Subject pauses prior to toe off
7. Hip extension on affected side	1 = Equal extension
	2 = Slight flexion
	3 = Marked flexion
Swing phase affected leg 8. External rotation during initial swing	1 = Same as unimpaired leg
	2 = Increased rotation: Externally rotates the leg < 45 degrees
	3 = Marked Rotation: Externally rotates the leg > 45 degrees
9. Circumduction at mid swing	1 = None
	2 = Moderate: Affected foot abducts up to one shoe width during swing
	3 = Marked: Affected foot circumducts more than one shoe width during swing
10. Hip hiking at mid swing	1 = None
	2 = Elevation
	3 = Vaults
11. Knee flexion from toe off to mid swing	1 = Normal (affected knee flexes equally to unaffected side)
	2a = Some (affected knee flexes, but less than unaffected knee)
	2b = Some (affected knee flexes, but more than unaffected knee)
	3a = Minimal (minimal flexion noted in affected knee (hardly visible)
	3b = Maximal (maximal flexion noted in affected knee (well visible)
	4 = None (knee remains in extension throughout swing)
12. Toe clearance	1 = Normal: Toe clears the floor throughout swing
	2 = Slight drag: Toe drags slightly at the beginning of swing phase
	3 = Marked: Toe drags during the majority of swing
13. Pelvic rotation at terminal swing	1 = Forward: The pelvis is rotated forward to prepare for heel strike
	2 = Neutral: Posture is erect with pelvis in neutral rotation
	3 = Retracted: Pelvis has marked lag behind the unaffected leg
Heel strike affected leg 14. Initial foot contact	1 = Heel strike
	2 = Foot flat: Foot lands with weight distributed over entire foot
	3 = No contact of heel: Foot lands on the lateral border of the foot or toes

**Figure 1:**
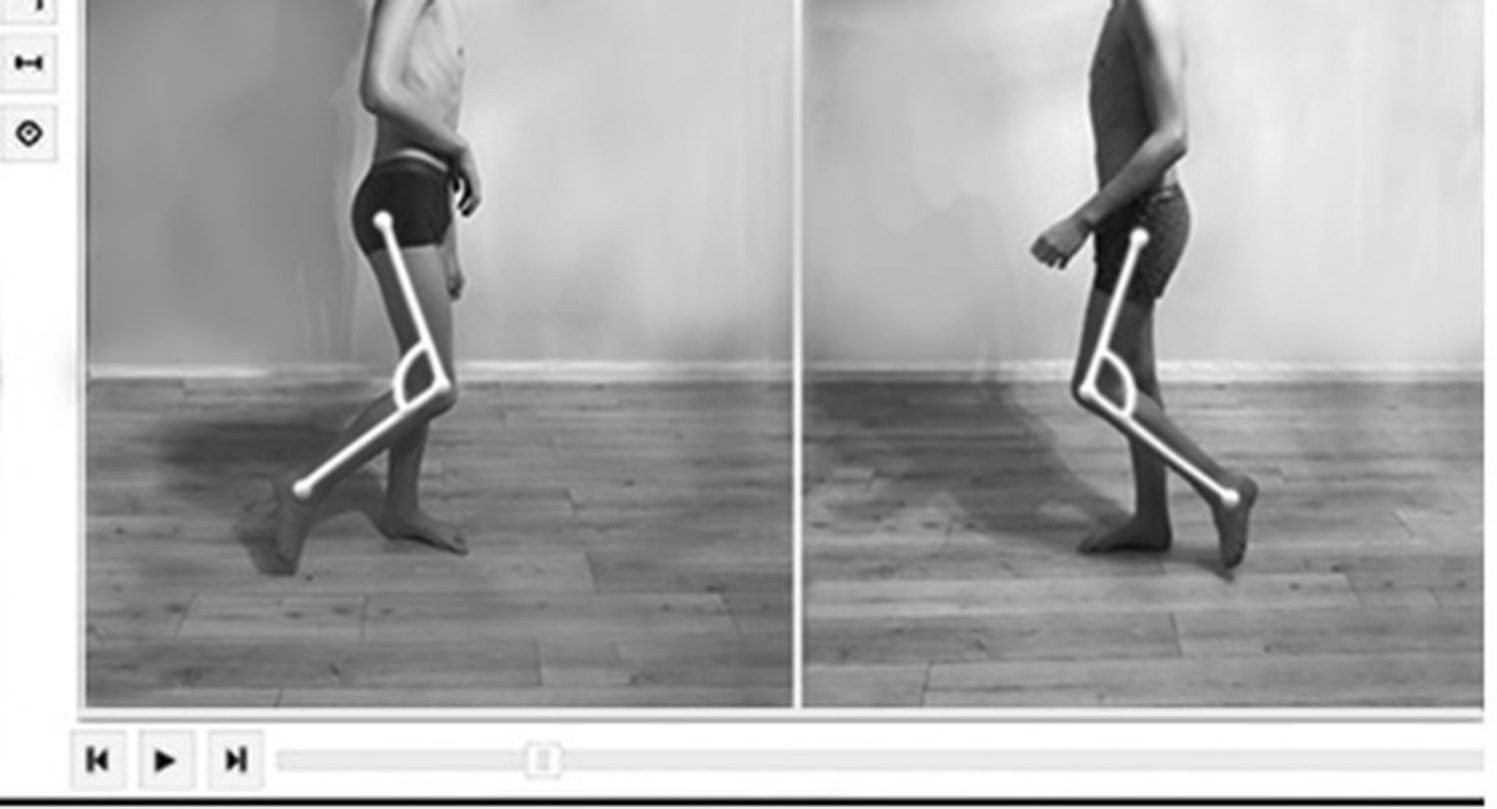
Screenshots of gait videos from the application software for computerised paediatric version of Wisconsin Gait Scale in child with right sided spastic hemiplegic cerebral palsy. The pictures show gait swing phase affected leg, knee flexion from toe off to mid swing. Two straight auxiliary lines are drawn on a freeze frame presenting a side view; these are located along the shank (head of the fibula and lateral malleolus) and along the thigh (trochanter of the femur and lateral condyle of femur). The angle created by these two lines is measured, and compared to the result identified for the unaffected leg. The rater examines the values of the angles, and determines the rating in the following way: affected knee flexes as much as the unaffected knee; affected knee flexes less than the unaffected knee; affected knee flexes more than the unaffected knee; minimal flexion observed in affected knee (hardly visible); maximal flexion observed in affected knee (well visible); knee remains in extension during the entire swing phase.

We designed a code, and a system consisting of an image analysis module, integrated with a database allowing us to process the data obtained from that module; the system also comprises another module which is used in reporting the data acquired during examinations. Model-View-Controller (MVC) design pattern was used in developing the interface, and the software can be operated in a standard PC environment (web browser). The system uses object-oriented programming, a relational database and scripting languages. Data processing is performed in the reporting module which also enables export of data to an Excel format. The software, based on a model comprising the items of the paediatric WGS, additionally utilises auxiliary lines between specific anthropometric points on the patient’s body, as well as angular values, duration and length of the specific gait phases, which enable acquisition of quantitative data corresponding to components of the paediatric WGS. The assessments were performed on the selected frames corresponding to the specific items of the paediatric version of WGS (angles and auxiliary lines were identified on the freeze frames matching the specific items of the paediatric WGS)—Fig. 1.

Gait of the children participating in the study was recorded in both the sagittal and the frontal plane using two synchronized video cameras (BTS Vixta system, BTS Bioengineering Corp.). During the trials children walked a distance of 10 m, at a self-selected speed, using their own orthopaedic aids, as needed. A minimum of 6 trials were registered, with three recordings focusing on the affected and three recordings focusing on the unaffected side. Gait analysis was carried out using the app, and after that the relevant data were retrieved from the software.

Inter-rater reliability of the new application software for computerised paediatric version of the WGS was evaluated by comparing results of assessments performed independently by three examiners, based on the video material acquired during the trials. Intra-rater reliability of the new application software for computerised paediatric WGS was determined by comparing results of two assessments of the same video materials, each performed in the same way two weeks apart (test–retest) by the same three examiners. Two week interval was thought to provide adequate time to prevent recall bias and ensure that the administration of the instruments across time was independent. The scoring was performed by physiotherapists with over 10-year experience in working with individuals with hemiplegic gait. The examiners were trained in the use and interpretation of the paediatric version of the WGS and had been involved in the earlier research. The examiners who evaluated participants' gait using computerised paediatric version of the WGS did not take the videos.

### Data analysis

The statistical analyses were performed using Statistica 13.1. The Shapiro–Wilk W-test was applied to examine the distributions of the investigated variables. The descriptive statistics computed for the numerical variables (items from computerised paediatric version of the WGS in points and for total score in points—the scale is a point system expressed by the number of points) included the mean, median, maximum and minimum values, the first as well as third quartile, and standard deviation. The Kruskal–Wallis analysis of variance (ANOVA) was used to compare the results of measurements 1 and 2 obtained by the three independent raters, and to assess differences in the median levels of numerical characteristics. The Wilcoxon signed-rank test was used to compare results identified by the same rater in measurement 1 and 2. The reliability coefficient was shown by the intra-class correlation coefficient (ICC). The two-way random effects ICC model for absolute agreement was used to establish intra- and inter-rater reliability^24^. We used ICC because it assesses agreement between two or more measurements of a quantitative variable (i.e. one that is expressed with a number), and as a result it applies to the paediatric version of the WGS, since it is expressed with a number of points assigned to the specific 14 items, and by a numerical total score, calculated by adding the points for the 14 items. The ICC makes it possible to assess whether and to what extent systematic and random errors affect measurement repeatability; its possible values are in the range of 0.00–1.00^25^. Reliability is thought to be poor if ICC values are lower than 0.40, fair if the values range from 0.40 to 0.59, good if the values are between 0.60 and 0.74, and excellent if the values are between 0.75 and 1.00^26^.

Bland and Altman analysis was also carried out in order to determine a quantitative estimate of how closely the values from two measurements are situated.

#### Sample size

The minimum sample size for the population investigated was computed using a sample size calculation feature (PLUS Module) included in Statistica 13.3 software, based on the following formula:

$$ {\text{N}}\min = \frac{{{\text{NP}}(\upalpha 2 \cdot {\text{f}}\left( {1 - {\text{f}}} \right))}}{{{\text{NP}} \cdot {\text{e}}2 + \upalpha 2 \cdot {\text{f}}\left( {1 - {\text{f}}} \right)}} $$

Nmin—minimum sample size, NP—size of the population from which the sample is drawn, α—level of confidence for the results, f—fraction size, e—expected maximum error.

## Results

The descriptive statistics for the total scores in the computerized paediatric version of WGS are shown in Table 3 (the descriptive statistics for the results/points in all the items of the computerized paediatric version of WGS, for three examiners in both measures are included in the supplementary materials—see Supplementary Table S1). The data presented in the next table reflect differences in assessments of the specific participants performed by the three examiners; there were no significant differences in the results obtained in measurements 1 and 2 (Table 4).

**Table 3:** Descriptive statistics for the computerized paediatric WGS.

Total score in the computerised modified paediatric WGS	Mean	Median	Min	Max	Quartile 1	Quartile 3	Standard deviation
Examiner 1 measurement 1	20.16	19.10	16.10	26.10	17.35	22.35	3.19
Examiner 1 measurement 2	20.07	19.10	16.10	26.10	18.10	23.10	3.01
Examiner 2 measurement 1	20.32	19.35	16.10	26.10	18.10	22.35	2.91
Examiner 2 measurement 2	20.32	20.10	16.10	26.10	18.10	22.10	2.78
Examiner 3 measurement 1	20.45	19.85	16.10	26.10	18.35	22.35	2.64
Examiner 3 measurement 2	20.74	20.35	16.10	26.10	19.10	22.10	2.67

**Table 4:** Differences in assessment of specific participants by three independent examiners in measurement 1 and measurements 2.

Items of computerised modified paediatric version of WGS	Measurement 1 p	Measurement 2 p
Stance phase affected leg 1. Use of hand-held gait aid	0.986	0.986
2. Stance time on affected side	0.819	0.792
3. Step length on unaffected side	0.923	0.566
4. Weight shift to affected side	0.593	0.706
5. Stance width	0.493	0.446
Toe off affected leg 6. Guardedness (pause prior to advancing affected leg)	0.236	0.367
7. Hip extension on affected side	0.999	0.990
Swing phase affected leg 8. External rotation during initial swing	0.957	0.643
9. Circumduction at mid swing	0.799	0.729
10. Hip hiking at mid swing	0.935	0.923
11. Knee flexion from toe off to mid swing	0.988	0.988
12. Toe clearance	0.956	0.956
13. Pelvic rotation at terminal swing	0.857	0.857
Heel strike affected leg 14. Initial foot contact	0.939	0.939
Total score	0.849	0.663

p—Probability index in Kruskal–Wallis ANOVA.

Subsequently, the scores obtained by the specific examiners were tested for correlations. The findings show no statistically significant differences between the scores awarded by the three examiners to the specific WGS items in measurement 1 and 2 (Table 5).

**Table 5:** Differences in assessment of the specific participants by the same examiner in measurement 1 and 2.

Items of computerised modified paediatric version of WGS	Examiner 1	Examiner 2	Examiner 3
Stance phase affected leg 1. Use of hand-held gait aid	1.000	1.000	1.000
2. Stance time on affected side	1.000	1.000	0.592
3. Step length on unaffected side	1.000	1.000	0.592
4. Weight shift to affected side	0.592	0.179	1.000
5. Stance width	1.000	1.000	0.179
Toe off affected leg 6. Guardedness (pause prior to advancing affected leg)	0.592	1.000	0.361
7. Hip extension on affected side	1.000	1.000	1.000
Swing phase affected leg 8. External rotation during initial swing	1.000	1.000	1.000
9. Circumduction at mid swing	1.000	1.000	1.000
10. Hip hiking at mid swing	1.000	1.000	1.000
11. Knee flexion from toe off to mid swing	1.000	1.000	1.000
12. Toe clearance	1.000	1.000	1.000
13. Pelvic rotation at terminal swing	1.000	1.000	1.000
Heel strike affected leg 14. Initial foot contact	1.000	1.000	1.000
Total score	0.678	0.208	0.650

p—Probability index in Wilcoxon signed-rank test.

Inter-rater reliability was excellent^26^, it was shown that ICC values were very high in measurement 1 (0.915–1.00) and 2 (0.896–1.00). Intra-rater reliability was also excellent^26^, reflected by ICC values in the range of 0.957–0.982.

In Bland–Altman analysis the 95% limits of agreement were in the range from 0 to 10.963. The intervals of agreement in the consecutive measurements ranged from 0.838 to 10.963—for total score in the case of examiner 1; from 0 to 2.195—for total score in the case of examiner 2; and from 0 to 2.491—for total score in the case of examiner 3 (Bland–Altman plots for all measurements are in supplementary materials—see Supplementary Figs. S1–S45).

## Discussion

The newly developed application software for the computerised paediatric version of the WGS, investigated in the current study, was shown to have very good intra- and inter-rater reliability. The mean scores acquired by three examiners in the specific measurement series carried out using the computerized paediatric WGS were very similar. Moreover, it was shown that ICC values were very high, which reflects a very high degree of agreement between the three examiners, and separately for each examiner in the two measurements. In our earlier study, we showed that the paediatric version of the WGS is a reliable tool for gait assessment in children with SHCP^21^. This was reflected by a very high ICC value as well as very good agreement and repeatability shown in Bland–Altman analysis^21^. In the present study we decided to go one step further, and develop a solution which would objectify the descriptive paediatric version of WGS, making it easier for researcher/clinician to accurately assess gait patters, and consequently making it possible to clarify disputable situations in the process of assessment based on the paediatric version of the WGS. As far as we know, the paediatric version of the WGS is the only scale making it possible to assess multivariate kinematic and spatiotemporal gait parameters in children with SHCP. Numerous related studies have investigated intra- and inter-observer reliability of scales used in observational assessment of gait in children with CP^8,11,27–31^, however, these tools focus only of evaluating kinematic parameters of gait. Furthermore, at present there are no computer-aided instruments designed to facilitate objective interpretation of scores acquired during observational gait assessment based on the paediatric WGS. The only scale designed specifically for children with spastic CP, and investigated for the same purpose is EVGS, for which smartphone slow-motion video technology and a motion analysis application were developed^22^. The related pilot study showed high inter- and intra-rater reliability of various components of the EVGS for two observers^22^.

In our study Bland–Altman analysis was performed for all the variables, in measurement 1 and 2, and for three examiners. Bland–Altman plot confirmed satisfactory agreement for all examiners. Klejman et al. also used Bland–Altman methods to examine the test–retest reliability of discrete gait parameters in children with CP^31^. In their analyses the limits of agreement were applied to determine the size of disagreement between baseline and retest values and were calculated by [d] ± 1.96 SDd, where d was defined as the difference score between baseline and retest, whereas SDd was the SD of the difference scores. They anticipated that 95% of the differences between baseline and retest for any person would be found between these limits^21^. The Bland–Altman analysis, however, did not use objective criteria, and should be interpreted in terms of clinical acceptability. In our case, before the study was initiated, we did not adopt any limits of agreement in the Bland–Altman analysis which for us would be satisfying from a clinical viewpoint. It was only after the analyses had been completed that we observed a certain tendency reflecting potential ranges of values, and we found that the 95% limits of agreement were in the range from 0 to 10.963. For us this is a point of reference, to be taken into account in practical use and in further research. Based on these findings, clinical acceptability can be assumed in the case of the narrowest 95% limits of agreement (in our case from 0 to slightly above 3), and these are related to the assessment of all the individual gait parameters. The widest 95% limit of agreement amounted to 10.963 however, it was only related to examiner 1 total score, rather than to individual evaluations of the specific gait parameters which are more precise compared to the global score. This may be evidence that the proposed computerised paediatric WGS enables more accurate assessment, which is also easier for the examiner, when it comes to the individual items related to gait parameters. As mentioned above, this wide 95% limit of agreement was found only once, for examiner 1 total score, whereas 95% limits of agreement for examiner 1 in the remaining 14 items assessed in the app were rather narrow, ranging from 0.838 to 3.35. For comparison, slightly better results in the range of 0–1.3052 and 0–1.2098 were identified in the case of examiner 2 and examiner 3, respectively. On the whole, these findings show that our results can be approached with optimism, suggesting that the proposed software application may effectively and reliably be used to perform gait analysis in children with hemiplegic CP. Moreover, it can be assumed that the computerised paediatric version of WGS presents practical advantages because it enables examiners to more easily make decisions in the process of WGS-based assessment in various cases.

In view of the above, it should be pointed out that the computerised paediatric WGS proposed in this study is characterised by high precision (agreement and reliability). Therefore, if further research shows that this tool can effectively monitor progress achieved by a specific patient (i.e., identify changes and variations occurring in course of the rehabilitation process), it could indeed be helpful for clinicians/physiotherapists in their daily assessment routines. Hence, it is necessary to continue evaluation of the proposed computerised paediatric version of WGS.

The current study presents certain limitations. One of these is related to the practical implications of the findings, since the proposed app is a “medical device” subject to legal requirements, as defined in the EU regulation 2017/745. Apart from that, further research is needed to examine the sensitivity of the tool to improvements resulting from specific therapies or rehabilitation programs. Furthermore, the validity the tool should be assessed e.g. by reference to the gold-standard, i.e., the instrumented gait analysis.

## Conclusions

The current findings provide evidence confirming very good intra- and inter-rater reliability of the proposed application software dedicated to the paediatric version of WGS. The observational gait scale, objectified through the new software, and enabling computer-aided use of the paediatric WGS, presents practical advantages for examiners since it facilitates decisions taken in the process of WGS-based assessment in children with SHCP.

## Supplementary Information

Supplementary Information 1.

Supplementary Information 2.

## Data Availability

The datasets used and/or analysed during the current study available from the corresponding author on reasonable request.
