# Supplementary material for: Inter- and intra-rater reliability of new application software for computerised paediatric version of Wisconsin Gait Scale
Source: Sci Rep. 2023 Mar 23;13:4757. doi: 10.1038/s41598-023-31436-8 (PMC10036550; doi:10.1038/s41598-023-31436-8)
Supplement: Supplementary file 1 — Supplementary Information 1. [file 41598_2023_31436_MOESM1_ESM.docx]

## Supplementary Figures

**Supplementary Figure S1.** Bland-Altman plot Examiner 1 (comparison of measurements 1 and 2) 1-use of hand-held gait aid (measurement 1) and 1-use of hand-held gait aid (measurement 2)

**Supplementary Figure S2.** Bland-Altman plot Examiner 1 (comparison of measurements 1 and 2) 2-stance time on affected side (measurement 1) and 2-stance time on affected side (measurement 2)

**Supplementary Figure S3.** Bland-Altman plot Examiner 1 (comparison of measurements 1 and 2) 3-step length on unaffected side (measurement 1) and 3-step length on unaffected side (measurement 2)

**Supplementary Figure S4.** Bland-Altman plot Examiner 1 (comparison of measurements 1 and 2) 4-weight shift to affected side (measurement 1) and 4-weight shift to affected side (measurement 2)

**Supplementary Figure S5.** Bland-Altman plot Examiner 1 (comparison of measurements 1 and 2) 5-stance width (measurement 1) and 5-stance width (measurement 2)

**Supplementary Figure S6.** Bland-Altman plot Examiner 1 (comparison of measurements 1 and 2) 6-guardedness (pause prior to advancing affected leg) (measurement 1) and 6-guardedness (pause prior to advancing affected leg) (measurement 2)

**Supplementary Figure S7.** Bland-Altman plot Examiner 1 (comparison of measurements 1 and 2) 7-hip extension on affected side (measurement 1) and 7-hip extension on affected side (measurement 2)

**Supplementary Figure S8.** Bland-Altman plot Examiner 1 (comparison of measurements 1 and 2) 8-external rotation during initial swing (measurement 1) and 8-external rotation during initial swing (measurement 2)

**Supplementary Figure S9.** Bland-Altman plot Examiner 1 (comparison of measurements 1 and 2) 9-circumduction at mid swing (measurement 1) and 9-circumduction at mid swing (measurement 2)

**Supplementary Figure S10.** Bland-Altman plot Examiner 1 (comparison of measurements 1 and 2) 10-hip hiking at mid swing (measurement 1) and 10-hip hiking at mid swing (measurement 2)

**Supplementary Figure S11.** Bland-Altman plot Examiner 1 (comparison of measurements 1 and 2) 11-knee flexion from toe off to mid swing (measurement 1) and 11-knee flexion from toe off to mid swing (measurement 2)

**Supplementary Figure S12.** Bland-Altman plot Examiner 1 (comparison of measurements 1 and 2) 12-toe clearance (measurement 1) and 12-toe clearance (measurement 2)

**Supplementary Figure S13.** Bland-Altman plot Examiner 1 (comparison of measurements 1 and 2) 13-pelvic rotation at terminal swing (measurement 1) and 13-pelvic rotation at terminal swing (measurement 2)

**Supplementary Figure S14.** Bland-Altman plot Examiner 1 (comparison of measurements 1 and 2) 14-initial foot contact (measurement 1) and 14-initial foot contact (measurement 2)

**Supplementary Figure S15.** Bland-Altman plot Examiner 1 (comparison of measurements 1 and 2) Total score (measurement 1) and Total score (measurement 2)

**Supplementary Figure S16.** Bland-Altman plot Examiner 2 (comparison of measurements 1 and 2) 1-use of hand-held gait aid (measurement 1) and 1-use of hand-held gait aid (measurement 2)

**Supplementary Figure S17.** Bland-Altman plot Examiner 2 (comparison of measurements 1 and 2) 2-stance time on affected side (measurement 1) and 2-stance time on affected side (measurement 2)

**Supplementary Figure S18.** Bland-Altman plot Examiner 2 (comparison of measurements 1 and 2) 3-step length on unaffected side (measurement 1) and 3-step length on unaffected side (measurement 2)

**Supplementary Figure S19.** Bland-Altman plot Examiner 2 (comparison of measurements 1 and 2) 4-weight shift to affected side (measurement 1) and 4-weight shift to affected side (measurement 2)

**Supplementary Figure S20.** Bland-Altman plot Examiner 2 (comparison of measurements 1 and 2) 5-stance width (measurement 1) and 5-stance width (measurement 2)

**Supplementary Figure S21.** Bland-Altman plot Examiner 2 (comparison of measurements 1 and 2) 6-guardedness (pause prior to advancing affected leg) (measurement 1) and 6-guardedness (pause prior to advancing affected leg) (measurement 2)

**Supplementary Figure S22.** Bland-Altman plot Examiner 2 (comparison of measurements 1 and 2) 7-hip extension on affected side (measurement 1) and 7-hip extension on affected side (measurement 2)

**Supplementary Figure S23.** Bland-Altman plot Examiner 2 (comparison of measurements 1 and 2) 8-external rotation during initial swing (measurement 1) and 8-external rotation during initial swing (measurement 2)

**Supplementary Figure S24.** Bland-Altman plot Examiner 2 (comparison of measurements 1 and 2) 9-circumduction at mid swing (measurement 1) and 9-circumduction at mid swing (measurement 2)

**Supplementary Figure S25.** Bland-Altman plot Examiner 2 (comparison of measurements 1 and 2) 10-hip hiking at mid swing (measurement 1) and 10-hip hiking at mid swing (measurement 2)

**Supplementary Figure S26.** Bland-Altman plot Examiner 2 (comparison of measurements 1 and 2) 11-knee flexion from toe off to mid swing (measurement 1) and 11-knee flexion from toe off to mid swing (measurement 2)

**Supplementary Figure S27.** Bland-Altman plot Examiner 2 (comparison of measurements 1 and 2) 12-toe clearance (measurement 1) and 12-toe clearance (measurement 2)

**Supplementary Figure S28.** Bland-Altman plot Examiner 2 (comparison of measurements 1 and 2) 13-pelvic rotation at terminal swing (measurement 1) and 13-pelvic rotation at terminal swing (measurement 2)

**Supplementary Figure S29.** Bland-Altman plot Examiner 2 (comparison of measurements 1 and 2) 14-initial foot contact (measurement 1) and 14-initial foot contact (measurement 2)

**Supplementary Figure S30.** Bland-Altman plot Examiner 2 (comparison of measurements 1 and 2) Total score (measurement 1) and Total score (measurement 2)

**Supplementary Figure S31.** Bland-Altman plot Examiner 3 (comparison of measurements 1 and 2) 1-use of hand-held gait aid (measurement 1) and 1-use of hand-held gait aid (measurement 2)

**Supplementary Figure S32.** Bland-Altman plot Examiner 3 (comparison of measurements 1 and 2) 2-stance time on affected side (measurement 1) and 2-stance time on affected side (measurement 2)

**Supplementary Figure S33.** Bland-Altman plot Examiner 3 (comparison of measurements 1 and 2) 3-step length on unaffected side (measurement 1) and 3-step length on unaffected side (measurement 2)

**Supplementary Figure S34.** Bland-Altman plot Examiner 3 (comparison of measurements 1 and 2) 4-weight shift to affected side (measurement 1) and 4-weight shift to affected side (measurement 2)

**Supplementary Figure S35.** Bland-Altman plot Examiner 3 (comparison of measurements 1 and 2) 5-stance width (measurement 1) and 5-stance width (measurement 2)

**Supplementary Figure S36.** Bland-Altman plot Examiner 3 (comparison of measurements 1 and 2) 6-guardedness (pause prior to advancing affected leg) (measurement 1) and 6-guardedness (pause prior to advancing affected leg) (measurement 2)

**Supplementary Figure S37.** Bland-Altman plot Examiner 3 (comparison of measurements 1 and 2) 7-hip extension on affected side (measurement 1) and 7-hip extension on affected side (measurement 2)

**Supplementary Figure S38.** Bland-Altman plot Examiner 3 (comparison of measurements 1 and 2) 8-external rotation during initial swing (measurement 1) and 8-external rotation during initial swing (measurement 2)

**Supplementary Figure S39.** Bland-Altman plot Examiner 3 (comparison of measurements 1 and 2) 9-circumduction at mid swing (measurement 1) and 9-circumduction at mid swing (measurement 2)

**Supplementary Figure S40.** Bland-Altman plot Examiner 3 (comparison of measurements 1 and 2) 10-hip hiking at mid swing (measurement 1) and 10-hip hiking at mid swing (measurement 2)

**Supplementary Figure S41.** Bland-Altman plot Examiner 3 (comparison of measurements 1 and 2) 11-knee flexion from toe off to mid swing (measurement 1) and 11-knee flexion from toe off to mid swing (measurement 2)

**Supplementary Figure S42.** Bland-Altman plot Examiner 3 (comparison of measurements 1 and 2) 12-toe clearance (measurement 1) and 12-toe clearance (measurement 2)

**Supplementary Figure S43.** Bland-Altman plot Examiner 3 (comparison of measurements 1 and 2) 13-pelvic rotation at terminal swing (measurement 1) and 13-pelvic rotation at terminal swing (measurement 2)

**Supplementary Figure S44.** Bland-Altman plot Examiner 3 (comparison of measurements 1 and 2) 14-initial foot contact (measurement 1) and 14-initial foot contact (measurement 2)

**Supplementary Figure S45.** Bland-Altman plot Examiner 3 (comparison of measurements 1 and 2) Total score (measurement 1) and Total score (measurement 2)
