# Supplementary material for: Inter- and intra-rater reliability of new application software for computerised paediatric version of Wisconsin Gait Scale
Source: Sci Rep. 2023 Mar 23;13:4757. doi: 10.1038/s41598-023-31436-8 (PMC10036550; doi:10.1038/s41598-023-31436-8)
Supplement: Supplementary file 2 — Supplementary Information 2. [file 41598_2023_31436_MOESM2_ESM.docx]

## Supplementary Tables

**Supplementary Table S1.** Descriptive statistics for the computerized paediatric WGS.

| Basic descriptive statistics | | | | | | | | |
| --- | --- | --- | --- | --- | --- | --- | --- | --- |
| Parameters (unit=points) | Examiner/measurement | Mean | Median | Min. | Max. | Quartile 1 | Quartile 3 | Standard deviation |
| STANCE PHASE AFFECTED LEG  1. use of hand-held gait aid | Examiner 1 measurement 1 | 0.64 | 0.60 | 0.60 | 1.80 | 0.60 | 0.60 | 0.22 |
|  | Examiner 1 measurement 2 | 0.64 | 0.60 | 0.60 | 1.80 | 0.60 | 0.60 | 0.22 |
|  | Examiner 2 measurement 1 | 0.64 | 0.60 | 0.60 | 1.80 | 0.60 | 0.60 | 0.22 |
|  | Examiner 2 measurement 2 | 0.64 | 0.60 | 0.60 | 1.80 | 0.60 | 0.60 | 0.22 |
|  | Examiner 3 measurement 1 | 0.64 | 0.60 | 0.60 | 1.80 | 0.60 | 0.60 | 0.22 |
|  | Examiner 3 measurement 2 | 0.64 | 0.60 | 0.60 | 1.80 | 0.60 | 0.60 | 0.22 |
| 2. stance time on affected  side | Examiner 1 measurement 1 | 1.68 | 2.00 | 1.00 | 3.00 | 1.00 | 2.00 | 0.70 |
|  | Examiner 1 measurement 2 | 1.65 | 2.00 | 1.00 | 3.00 | 1.00 | 2.00 | 0.71 |
|  | Examiner 2 measurement 1 | 1.61 | 2.00 | 1.00 | 3.00 | 1.00 | 2.00 | 0.67 |
|  | Examiner 2 measurement 2 | 1.58 | 2.00 | 1.00 | 3.00 | 1.00 | 2.00 | 0.62 |
|  | Examiner 3 measurement 1 | 1.61 | 2.00 | 1.00 | 3.00 | 1.00 | 2.00 | 0.67 |
|  | Examiner 3 measurement 2 | 1.61 | 2.00 | 1.00 | 3.00 | 1.00 | 2.00 | 0.67 |
| 3. step length on unaffected side | Examiner 1 measurement 1 | 1.13 | 1.00 | 1.00 | 2.00 | 1.00 | 1.00 | 0.34 |
|  | Examiner 1 measurement 2 | 1.16 | 1.00 | 1.00 | 2.00 | 1.00 | 1.00 | 0.37 |
|  | Examiner 2 measurement 1 | 1.13 | 1.00 | 1.00 | 2.00 | 1.00 | 1.00 | 0.34 |
|  | Examiner 2 measurement 2 | 1.13 | 1.00 | 1.00 | 2.00 | 1.00 | 1.00 | 0.34 |
|  | Examiner 3 measurement 1 | 1.23 | 1.00 | 1.00 | 2.00 | 1.00 | 1.00 | 0.43 |
|  | Examiner 3 measurement 2 | 1.29 | 1.00 | 1.00 | 2.00 | 1.00 | 2.00 | 0.46 |
| 4. weight shift to affected side | Examiner 1 measurement 1 | 1.90 | 2.00 | 1.00 | 3.00 | 1.00 | 2.00 | 0.65 |
|  | Examiner 1 measurement 2 | 1.74 | 2.00 | 1.00 | 3.00 | 1.00 | 2.00 | 0.51 |
|  | Examiner 2 measurement 1 | 1.84 | 2.00 | 1.00 | 3.00 | 1.00 | 2.00 | 0.58 |
|  | Examiner 2 measurement 2 | 1.87 | 2.00 | 1.00 | 3.00 | 2.00 | 2.00 | 0.56 |
|  | Examiner 3 measurement 1 | 1.81 | 2.00 | 1.00 | 3.00 | 1.00 | 2.00 | 0.54 |
|  | Examiner 3 measurement 2 | 1.84 | 2.00 | 1.00 | 3.00 | 2.00 | 2.00 | 0.52 |
| 5. stance width | Examiner 1 measurement 1 | 1.26 | 1.00 | 1.00 | 3.00 | 1.00 | 1.00 | 0.51 |
|  | Examiner 1 measurement 2 | 1.32 | 1.00 | 1.00 | 3.00 | 1.00 | 2.00 | 0.54 |
|  | Examiner 2 measurement 1 | 1.35 | 1.00 | 1.00 | 3.00 | 1.00 | 2.00 | 0.55 |
|  | Examiner 2 measurement 2 | 1.35 | 1.00 | 1.00 | 3.00 | 1.00 | 2.00 | 0.55 |
|  | Examiner 3 measurement 1 | 1.35 | 1.00 | 1.00 | 3.00 | 1.00 | 2.00 | 0.55 |
|  | Examiner 3 measurement 2 | 1.42 | 1.00 | 1.00 | 2.00 | 1.00 | 2.00 | 0.50 |
| TOE OFF AFFECTED LEG  6. guardedness (pause prior to advancing affected leg) | Examiner 1 measurement 1 | 1.16 | 1.00 | 1.00 | 2.00 | 1.00 | 1.00 | 0.37 |
|  | Examiner 1 measurement 2 | 1.19 | 1.00 | 1.00 | 2.00 | 1.00 | 1.00 | 0.40 |
|  | Examiner 2 measurement 1 | 1.26 | 1.00 | 1.00 | 2.00 | 1.00 | 2.00 | 0.44 |
|  | Examiner 2 measurement 2 | 1.26 | 1.00 | 1.00 | 2.00 | 1.00 | 2.00 | 0.44 |
|  | Examiner 3 measurement 1 | 1.32 | 1.00 | 1.00 | 3.00 | 1.00 | 2.00 | 0.54 |
|  | Examiner 3 measurement 2 | 1.32 | 1.00 | 1.00 | 3.00 | 1.00 | 2.00 | 0.54 |
| 7. hip extension on affected side | Examiner 1 measurement 1 | 1.58 | 2.00 | 1.00 | 3.00 | 1.00 | 2.00 | 0.62 |
|  | Examiner 1 measurement 2 | 1.58 | 2.00 | 1.00 | 3.00 | 1.00 | 2.00 | 0.62 |
|  | Examiner 2 measurement 1 | 1.61 | 2.00 | 1.00 | 3.00 | 1.00 | 2.00 | 0.62 |
|  | Examiner 2 measurement 2 | 1.58 | 2.00 | 1.00 | 3.00 | 1.00 | 2.00 | 0.56 |
|  | Examiner 3 measurement 1 | 1.61 | 2.00 | 1.00 | 3.00 | 1.00 | 2.00 | 0.56 |
|  | Examiner 3 measurement 2 | 1.61 | 2.00 | 1.00 | 3.00 | 1.00 | 2.00 | 0.56 |
| SWING PHASE AFFECTED LEG  8. external rotation during initial swing | Examiner 1 measurement 1 | 1.48 | 1.00 | 1.00 | 2.00 | 1.00 | 2.00 | 0.51 |
|  | Examiner 1 measurement 2 | 1.48 | 1.00 | 1.00 | 2.00 | 1.00 | 2.00 | 0.51 |
|  | Examiner 2 measurement 1 | 1.52 | 2.00 | 1.00 | 2.00 | 1.00 | 2.00 | 0.51 |
|  | Examiner 2 measurement 2 | 1.48 | 1.00 | 1.00 | 2.00 | 1.00 | 2.00 | 0.51 |
|  | Examiner 3 measurement 1 | 1.52 | 2.00 | 1.00 | 2.00 | 1.00 | 2.00 | 0.51 |
|  | Examiner 3 measurement 2 | 1.58 | 2.00 | 1.00 | 2.00 | 1.00 | 2.00 | 0.50 |
| 9. circumduction at mid swing | Examiner 1 measurement 1 | 1.58 | 2.00 | 1.00 | 2.00 | 1.00 | 2.00 | 0.50 |
|  | Examiner 1 measurement 2 | 1.58 | 2.00 | 1.00 | 2.00 | 1.00 | 2.00 | 0.50 |
|  | Examiner 2 measurement 1 | 1.61 | 2.00 | 1.00 | 2.00 | 1.00 | 2.00 | 0.50 |
|  | Examiner 2 measurement 2 | 1.65 | 2.00 | 1.00 | 2.00 | 1.00 | 2.00 | 0.49 |
|  | Examiner 3 measurement 1 | 1.58 | 2.00 | 1.00 | 2.00 | 1.00 | 2.00 | 0.50 |
|  | Examiner 3 measurement 2 | 1.68 | 2.00 | 1.00 | 3.00 | 1.00 | 2.00 | 0.54 |
| 10. hip hiking at mid swing | Examiner 1 measurement 1 | 1.55 | 2.00 | 1.00 | 3.00 | 1.00 | 2.00 | 0.57 |
|  | Examiner 1 measurement 2 | 1.52 | 2.00 | 1.00 | 2.00 | 1.00 | 2.00 | 0.51 |
|  | Examiner 2 measurement 1 | 1.55 | 2.00 | 1.00 | 3.00 | 1.00 | 2.00 | 0.57 |
|  | Examiner 2 measurement 2 | 1.58 | 2.00 | 1.00 | 3.00 | 1.00 | 2.00 | 0.56 |
|  | Examiner 3 measurement 1 | 1.58 | 2.00 | 1.00 | 3.00 | 1.00 | 2.00 | 0.56 |
|  | Examiner 3 measurement 2 | 1.55 | 2.00 | 1.00 | 2.00 | 1.00 | 2.00 | 0.51 |
| 11. knee flexion from toe off to mid swing | Examiner 1 measurement 1 | 1.23 | 1.50 | 0.75 | 2.25 | 0.75 | 1.50 | 0.46 |
|  | Examiner 1 measurement 2 | 1.23 | 1.50 | 0.75 | 2.25 | 0.75 | 1.50 | 0.46 |
|  | Examiner 2 measurement 1 | 1.23 | 1.50 | 0.75 | 2.25 | 0.75 | 1.50 | 0.46 |
|  | Examiner 2 measurement 2 | 1.23 | 1.50 | 0.75 | 2.25 | 0.75 | 1.50 | 0.46 |
|  | Examiner 3 measurement 1 | 1.23 | 1.50 | 0.75 | 2.25 | 0.75 | 1.50 | 0.46 |
|  | Examiner 3 measurement 2 | 1.23 | 1.50 | 0.75 | 2.25 | 0.75 | 1.50 | 0.46 |
| 12. toe clearance | Examiner 1 measurement 1 | 1.10 | 1.00 | 1.00 | 2.00 | 1.00 | 1.00 | 0.30 |
|  | Examiner 1 measurement 2 | 1.10 | 1.00 | 1.00 | 2.00 | 1.00 | 1.00 | 0.30 |
|  | Examiner 2 measurement 1 | 1.10 | 1.00 | 1.00 | 2.00 | 1.00 | 1.00 | 0.30 |
|  | Examiner 2 measurement 2 | 1.10 | 1.00 | 1.00 | 2.00 | 1.00 | 1.00 | 0.30 |
|  | Examiner 3 measurement 1 | 1.10 | 1.00 | 1.00 | 2.00 | 1.00 | 1.00 | 0.30 |
|  | Examiner 3 measurement 2 | 1.10 | 1.00 | 1.00 | 2.00 | 1.00 | 1.00 | 0.30 |
| 13. pelvic rotation at terminal swing | Examiner 1 measurement 1 | 1.90 | 2.00 | 1.00 | 3.00 | 1.00 | 3.00 | 0.87 |
|  | Examiner 1 measurement 2 | 1.90 | 2.00 | 1.00 | 3.00 | 1.00 | 3.00 | 0.87 |
|  | Examiner 2 measurement 1 | 1.90 | 2.00 | 1.00 | 3.00 | 1.00 | 3.00 | 0.87 |
|  | Examiner 2 measurement 2 | 1.90 | 2.00 | 1.00 | 3.00 | 1.00 | 3.00 | 0.87 |
|  | Examiner 3 measurement 1 | 1.90 | 2.00 | 1.00 | 3.00 | 1.00 | 3.00 | 0.87 |
|  | Examiner 3 measurement 2 | 1.90 | 2.00 | 1.00 | 3.00 | 1.00 | 3.00 | 0.87 |
| HEEL STRIKE AFFECTED LEG  14. initial foot contact | Examiner 1 measurement 1 | 1.97 | 2.00 | 1.00 | 3.00 | 1.00 | 2.00 | 0.71 |
|  | Examiner 1 measurement 2 | 1.97 | 2.00 | 1.00 | 3.00 | 1.00 | 2.00 | 0.71 |
|  | Examiner 2 measurement 1 | 1.97 | 2.00 | 1.00 | 3.00 | 1.00 | 2.00 | 0.71 |
|  | Examiner 2 measurement 2 | 1.97 | 2.00 | 1.00 | 3.00 | 1.00 | 2.00 | 0.71 |
|  | Examiner 3 measurement 1 | 1.97 | 2.00 | 1.00 | 3.00 | 1.00 | 2.00 | 0.71 |
|  | Examiner 3 measurement 2 | 1.97 | 2.00 | 1.00 | 3.00 | 1.00 | 2.00 | 0.71 |
| Total score | Examiner 1 measurement 1 | 2.16 | 19.10 | 16.10 | 26.10 | 17.35 | 22.35 | 3.19 |
|  | Examiner 1 measurement 2 | 2.07 | 19.10 | 16.10 | 26.10 | 18.10 | 23.10 | 3.01 |
|  | Examiner 2 measurement 1 | 2.32 | 19.35 | 16.10 | 26.10 | 18.10 | 22.35 | 2.91 |
|  | Examiner 2 measurement 2 | 2.32 | 20.10 | 16.10 | 26.10 | 18.10 | 22.10 | 2.78 |
|  | Examiner 3 measurement 1 | 2.45 | 19.85 | 16.10 | 26.10 | 18.35 | 22.35 | 2.64 |
|  | Examiner 3 measurement 2 | 2.74 | 20.35 | 16.10 | 26.10 | 19.10 | 22.10 | 2.67 |

.
